# Global Seropositivity of Swine Leptospirosis: Systematic Review and Meta-Analysis

**DOI:** 10.3390/tropicalmed8030158

**Published:** 2023-03-05

**Authors:** Hosaneide Gomes de Araújo, Clécio Henrique Limeira, Vitória Viviane Ferreira de Aquino, Vinícius Longo Ribeiro Vilela, Clebert José Alves, Severino Silvano dos Santos Higino, Carolina de Sousa Américo Batista Santos, Sérgio Santos de Azevedo

**Affiliations:** 1 Academic Unit of Veterinary Medicine (UAMV), Federal University of Campina Grande (UFCG), Patos 58708-110, Brazil; 2 Department of Veterinary Medicine, Federal Institute of Paraíba (IFPB), Sousa 58814-000, Brazil

**Keywords:** *Leptospira* spp., pig, serology, epidemiology, control, one health

## Abstract

Leptospirosis is a major threat to public health worldwide; however, there is no study focused on global seropositivity in pigs. In this study, we grouped publications and performed a systematic review with meta-analysis to gather data related to swine leptospirosis seropositivity published globally. The search method initially used returned a total of 1183 results, of which 20 met all predefined criteria and were therefore included in this review. Meta-analysis with general data was performed and a combined seropositivity of 21.95% was found. Seropositivity was 36.40% in South America, 34.05% in North America, 22.18% in Africa, 17.40% in Oceania, 13.30% in Europe and 13.36% in Asia. The results suggest that there is high seropositivity for leptospirosis in pigs worldwide. Information compiled from this research is relevant to understanding the spread of leptospirosis globally. It is expected that these indicators will contribute to a better understanding of the epidemiology of the disease with a focus on its control and, consequently, on the reduction of cases in the human and animal population.

## 1. Introduction

Leptospirosis is an important zoonotic disease with global distribution, caused by pathogenic spirochete bacteria of the genus *Leptospira*, of which more than 260 serovars have been identified in recent years [1]. It is endemic in tropical countries due to geoclimatic and social conditions, which favor disease transmission and contribute to its increasing incidence [2].

Leptospires infect several animal species, including cattle, dogs, horses, pigs, small ruminants and wild animals [3]. Most animals are asymptomatically infected, with leptospires located in the kidneys and eliminated in the environment, and under appropriate environmental conditions can survive for weeks or months. In pigs, there are records that the pathogen can cause fetal infection in the acute phase and genital lesions in the chronic phase of the disease [4,5,6].

The main sources of infection are rodents and small marsupials, cattle, pigs and dogs [7]. People who live in rural areas are more exposed to risk, especially in tropical countries, where they are in close contact with environments inhabited by sources of infection [8]. Transmission can occur indirectly through contact with contaminated water or soil or through direct contact with the urine of infected animals [9].

Ospina-Pinto and Hernández-Rodríguez [10] isolated leptospires from urine and water from a population of swine and identified that the serovar Canicola was present in both types of samples, and the authors suggested that the bacterium could be transmitted in the animal–environment interface, mainly in the piglet stage. Other similar studies aiming at the isolation of *Leptospira* spp. were reported in pigs and other animals, water and soil in Nicaragua, and *L. weilii* and *L. interrogans* were recovered from pigs and other species, water and humans in Thailand [11,12]. 

The microscopic agglutination test (MAT) is considered a reference in the serological diagnosis of leptospirosis, mainly for epidemiological studies, as it is able to test for several serovars that represent different serogroups at once. The principle of the technique is based on the antigen–antibody reaction and it detects both IgM and IgG antibody classes [13]. With this technique, it is often not possible to identify the infecting serogroup, as there is usually cross-reaction between serovars that belong to the same serogroup [14]. In this regard, the ranking technique has been pointed out as a suitable method of interpreting the results obtained by MAT in order to refine the data, reducing the occurrence of cross reactions between the serogroups, and it can be useful in the MAT for predicting the most likely infecting serogroup of Leptospira, and can be applied especially in epidemiological studies involving herds [13].

The use of vaccines can make it difficult to interpret serological tests that are widely used for herd health monitoring and surveillance because they are cost-effective and provide evidence of exposure [15,16]. Previous studies claim that MAT titers are very low with vaccination and are much higher in naturally infected pigs [15,16], and for this reason, MAT titers of 1:100 or more against one or more serovars are generally considered significant [16].

The control of leptospirosis is generally difficult and differs for each host species and infectious serogroup of *Leptospira* spp. Thus, the knowledge of the serogroups prevalent for each host is important from the control point of view [17], which raises not only the identification of serogroups, but the dynamics of the infectious agent in a holistic way. 

Pigs are deemed reservoir hosts for the Muenchen, Pomona and Tarassovi Mitis serovars, and can also get infected by other serovars, mainly Icterohaemorrhagiae, Canicola and Hardjo [18]. The Icterohaemorrhagiae, Grippotyphosa and Tarassovi serogroups are involved in the most commonly identified incidental infections in swine [19,20]. A complex epidemiology, wide range of susceptible hosts and reservoirs, intricate ecology and limitation in the cultivation of the bacterium make it difficult to diagnose the disease in farm animals, especially in its subclinical forms [21]. It is important to emphasize that swine leptospirosis is relevant and that there is no systematic review with meta-analysis published so far on seropositivity of the infection in pigs; therefore, this review aimed to determine the global prevalence of swine leptospirosis. 

## 2. Materials and Methods

### 2.1. Data Sources

A descriptive exploratory study through a systematic review of the literature with meta-analysis was carried out. This investigation was operationalized according to the Preferred Reporting Items for Systematic Reviews and Meta-Analyses (PRISMA) protocol, through the following steps: identification, selection, eligibility and inclusion [22]. The survey was registered on the International Prospective Registry of Systematic Reviews (PROSPERO) platform with the identification number 201293.

To find published studies related to leptospirosis in pigs, five scientific databases were consulted: Science Direct, Medline, SciELO, LILACS and PubMed. The descriptors used in the search process were “prevalence”, “leptospirosis” and “swine” and to combine these descriptors, the logical operator “AND” was used and all publications found were exported in a “BibTex” file to the bibliographic manager Mendeley^®^, from which duplicates were excluded, allowing the reading of the titles and abstracts of primary studies by four researchers, independently. There was no language restriction or year of publication, and the searches were carried out on 30 June 2022.

### 2.2. Criteria for Data Inclusion and Extraction

The inclusion criteria for a more detailed analysis were (i) performing serology using microscopic agglutination test (MAT); (ii) scientific articles that contained data on seropositivity of leptospirosis in swine; (iii) cross-sectional or cohort studies. After an analysis of the articles, the ones that did not meet these criteria (for example, outbreak reports) were excluded [23].

For the selection of studies and data extraction, the Mendeley software version v1.19.8 (Mendeley Ltd., London, UK) was used. After removing duplicate records, three researchers (Araújo HG, Limeira HC and Vilela VLR) selected articles by title and abstract, obeying the predefined inclusion criteria. The disagreed cases were resolved by consensus or decision of a fourth researcher (Azevedo SS). Then, the complete texts were gathered for evaluation. In all published studies, the materials and methods section was carefully read and analyzed.

The following data were extracted from the studies: author; year; country; continent; sample size; number of positive pigs; diagnostic method. In cases of disagreement, the decision was made by consensus.

### 2.3. Data Analysis

Using the seropositivity of leptospirosis infection as the primary outcome, a meta-analysis was carried out with all primary studies included, without subdivisions, in which the presence of heterogeneity was observed. Thus, it was decided to apply another meta-analysis, dividing the studies into subgroups, according to the continent of research and the sample size, in order to identify the origin of the heterogeneity.

Heterogeneity was assessed by Cochran’s Q test and quantified by Higgins and Thompson’s I^2^ test. The combined seropositivity estimates and the 95% confidence interval (CI) were calculated based on the random effects model by the inverse of variance, using the DerSimonian–Laird method. The presence of publication bias was analyzed by viewing the funnel plot and applying the Egger test [24]. All analyses were performed on R environment [25] and RStudio interface (version 1.1.463), using the statistical packages “meta” [26] and “metafor” [27].

## 3. Results

**Figure 1:**
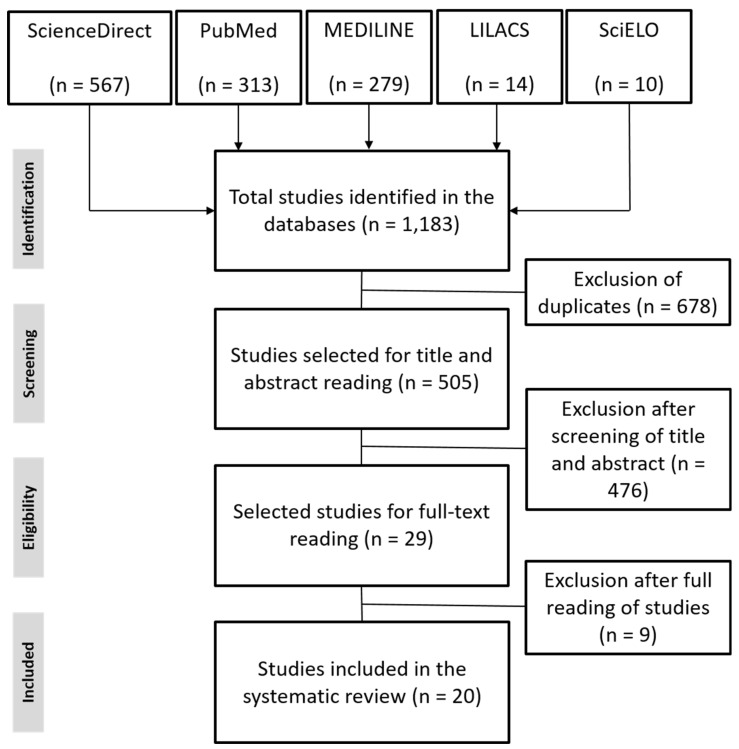
Flowchart based on the PRISMA protocol on evidence selection.

The search method used initially returned a total of 1183 results, of which 20 met all predefined criteria and were therefore included in this review. Following the recommendation of the PRISMA group, the search strategies and reasons for exclusion used in the respective databases are specified in the flowchart shown in Figure 1.

The 20 studies were carried out in several countries on six different continents: South America (*n* = 6), Europe (*n* = 3), Oceania (*n* = 4), Asia (*n* = 4), North America (*n* = 2) and Africa (*n* = 1), and the main information and data used to perform the meta-analysis of the seropositivity of swine in leptospirosis are shown in Table 1.

**Figure 2:**
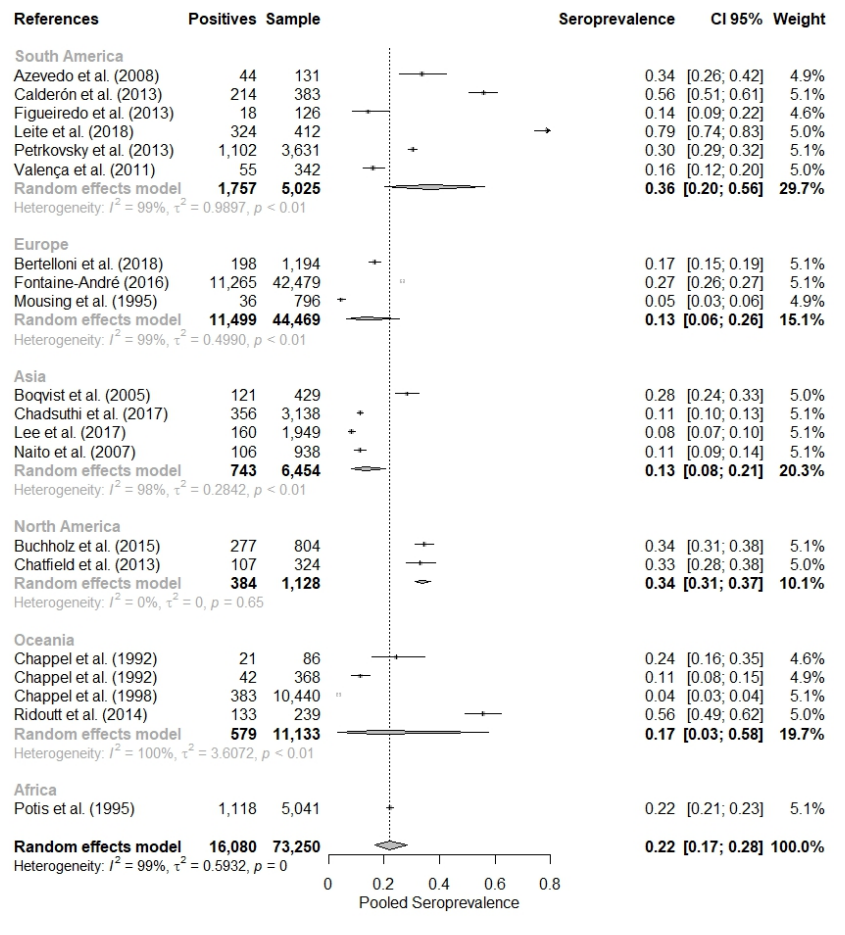
Combined evaluation of twenty studies on the seropositivity of leptospirosis in pigs [28,29,30,31,32,33,34,35,36,37,38,39,40,41,42,43,44,45,46,47].

Initially, a meta-analysis with the general data was performed, and a combined seropositivity of 21.95% (95% CI = 16.65–28.37%) was found, but with the presence of heterogeneity identified by the Cochran Q test (*p* < 0.01) and classified as high heterogeneity by the Higgins and Thompson test (I2 = 99.45%) (Figure 2).

When evaluating the meta-analysis by continents (Table 2), it was observed that the seropositivity of leptospirosis in swine was higher in South America (36.40%; 95% CI = 20.36–56.16%), followed by North America, 34.05% (95% CI = 31.34–36.86%), Africa As default, R environment include commas for number with four or more digits. We have provided (22.18%; 95% CI = 21.05–23.35%), Oceania (17.40%; 95% CI = 3.15–57.70%), Europe (13.30%; 95% CI = 6.40–25.62%) and Asia (13.36%; 95% CI = 8.32–20.75%).

**Figure 3:**
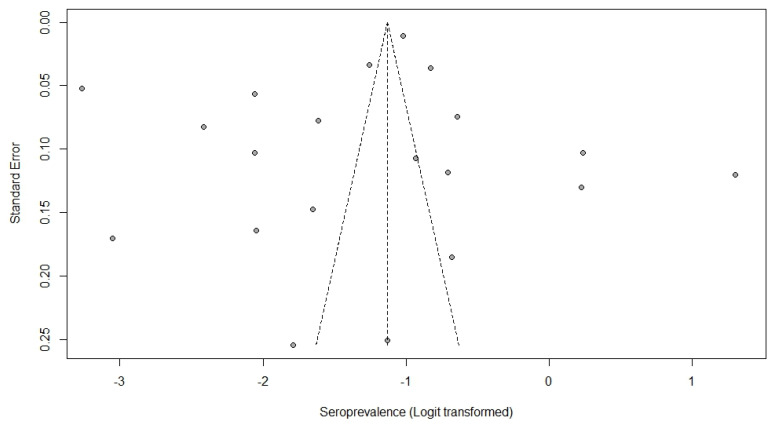
Funnel plot showing the distribution of studies (points) according to prevalence and standard errors.

Egger’s test was calculated, resulting in a value of *p* = 0.4256, indicating no bias from small studies. The visualization of the funnel plot (Figure 3) indicates an asymmetric distribution of the points (representing the 20 studies included in the meta-analysis), suggesting the presence of publication bias.

## 4. Discussion

This systematic review allowed us to identify and summarize the evidence published in studies that aimed to describe the seropositivity of leptospirosis in pigs. A high level of heterogeneity was observed in the combined estimate, which was expected since all studies included in the review were observational surveys, which are subject to several systematic biases [48]. Cross-sectional surveys are the most frequently designed observational studies in veterinary epidemiology, likely because they are rapid, inexpensive and of moderate difficulty. However, it is important to be aware of the primary limitation of cross-sectional studies, i.e., there is generally no evidence of a temporal relationship because exposure and outcome are simultaneously assessed, and without longitudinal data, it is not possible to establish a true cause and effect relationship [49,50].

Articles indexed in five important databases on serological studies of leptospirosis in pigs were analyzed, after the application of the inclusion criteria and, surprisingly, a low number of indexed articles were identified, since there was a wide search without time limitation. These results are relevant and can be used to alert about the importance of conducting serological surveys on swine leptospirosis, since it was mentioned as one of the most frequent infectious diseases in animals, presenting as subclinical or clinical infection [3,23,51,52]. This reinforced that in animal production, *Leptospira* spp. infection causes reproductive problems such as abortions, stillbirths and weak piglets, which affects animal welfare and the production chain with consequent loss of production and income [53].

Egger’s test was calculated, resulting in a value of *p* = 0.4256, indicating no bias from small studies. Thus, the relative asymmetry observed in the funnel plot may be related to the high heterogeneity found in the meta-analysis [54]. 

**Figure 4:**
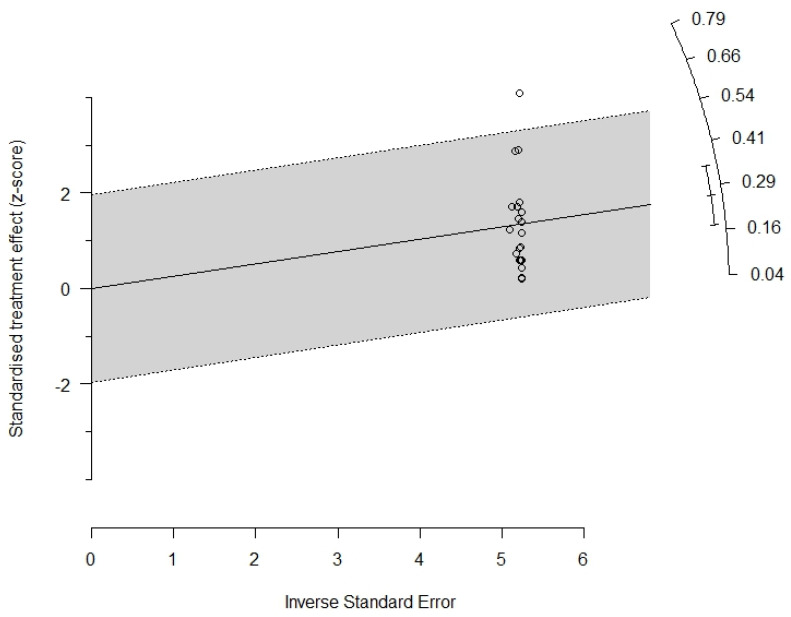
Radial plot (Galbraith plots).

The radial graph (Galbraith plot) included in the manuscript (Figure 4) showed only one study outside the confidence interval, considered an outlier [38], but its exclusion did not significantly change the heterogeneity. The radial graph also shows that the studies have good accuracy far from the origin of the axes, but with a dispersed vertical distribution, which indicates high heterogeneity. Thus, analysis of the Egger test in conjunction with the Galbraith plot provides evidence that funnel asymmetry may be related to other factors, and not the presence of bias in small studies, such as high heterogeneity.

Studies carried out in South America showed seropositivity from 14.3% to 78.6%, with a predominance of serogroup Icterohaemorrhagiae, followed by Pomona and Autumnalis. The difference between the seropositivity values can be explained by the location of the studies and the type of breeding adopted by the producers. Valença et al. [47], who established 16.1% seropositivity, carried out their survey on technically swine farms in the state of Alagoas, Brazil, while Leite et al. [41], who reported 78.6% seropositivity, developed the study in non-technified farms in Mossoró, in the state of Rio Grande do Norte, Brazil. These results demonstrate that technical care applied correctly is effective in reducing the infectious agent in swine production. Valença et al. [47] also pointed out that factors such as lack of quarantine and artificial insemination represented flaws that may have been responsible for contamination of the herd by Leptospira spp. Studies that investigate not only seropositivity, but also provide information on the risk factors associated with seropositivity and transmission of leptospirosis are especially relevant, as in addition to contributing to the detection of the most prevalent species, they reveal data that can be used to correct flaws in the management.

The authors Azevedo et al. [28] and Figueiredo et al. [38] reported seropositivity rates of 33.6% and 14.6%, respectively, in studies carried out in the same slaughterhouse in the city of Patos, in the state of Paraíba, Brazil. Slaughterhouses are important sources of information, but they do not allow the collection of sufficient data to obtain information to enable a more in-depth epidemiological assessment. Studies like these are of great relevance, as they identify the presence of the pathogen, but for a more broad epidemiological evaluation, studies must be carried out on the properties in order to collect data that can contribute to the effective control of the disease in the region.

Petrakovsky et al. [44], in a study carried out on throughout Argentina, detected the presence of anti-*Leptospira* spp. antibodies in pigs, without clinical symptoms. The location of the farms and the samples were chosen proportionally to those registered in each province covering the entire country, and 30% of the samples tested were positive for MAT. Most of these samples showed coagglutinins for two or three serovars. In the sera titration, the percentage of positives was the same, and the most prevalent serovars were Icterohaemorrhagiae and Castellonis. Ninety percent of the samples had a final titer of 1:200. The existence of positive sera in all provinces indicates contact of animals with leptospira throughout the country. This type of study shows the importance of epidemiological investigation, as it presents data that make it possible to minimize the damage caused by the causative agent of leptospirosis in swine herds with the possibility of adequate intervention and prevention.

Calderón et al. [32] sought to determine the seroprevalence in humans and animals and to isolate *Leptospira interrogans* sensu lato in domestic animals, rodents and water sources. The study was carried out in a tropical area of the middle Sinú in Córdoba, Colombia. In a prospective descriptive study, blood and urine from pigs and dogs, sera from rural workers, renal tissues of rodents, and water samples from environmental sources were collected. The seroprevalence found was 55.9% in pigs, 35.2% in dogs and 75.8% in humans; no antibodies were detected and no leptospire was isolated from rodent kidneys. Seven sensu lato strains of *L. interrogans* were isolated: three from pigs, two from dogs and two from water. The seroprevalence detected in pigs, dogs and humans, concomitant with the isolation of strains, suggests that in Córdoba, there may be transmission between animals, the environment and humans. This research investigated the environment, animals and human beings, presenting an important research model by gathering information on the dynamics of leptospirosis, taking into account the concept of unique health and directing, through the evidence found, effective intervention measures to reduce the impact caused by leptospirosis.

In North America, a combined seropositivity of 34.05% (95% CI = 31.34–36.86%) was identified in two studies that investigated the presence of leptospirosis in feral pig populations, but no work using the search method of this research was found with domestic pigs. Chatfield et al. [37] sought to establish the preliminary seroprevalence of leptospirosis exposure in wild pigs in Florida, after noting that there were no published studies to date. They opportunistically collected blood samples from 158 male and 166 female wild pigs from controlled hunts and by licensed hunters in north, central and south Florida. The samples were then analyzed using the microscopic agglutination test (MAT) and 33% of the total samples (107/324) were positive for at least one serovar, and 46% of these were positive for multiple serovars. These initial data indicate that there is a significant possibility that feral pigs play a greater role in the complex etiology of leptospirosis in Florida than historically estimated, and that additional investigations should be conducted to reinforce the findings of this research.

Buchholz et al. [31] researched the seroprevalence of leptospirosis in wild pigs in Hawaii, USA, from 2007 to 2009, in blood samples collected opportunistically. Using the microscopic agglutination test, they found antibody titers ≥1:100 to leptospires in 272 (33.8%) of 804 wild pigs. The serovars with the most frequent reaction to swine sera were Icterohaemorrhagiae (serogroup Icterohaemorrhagiae) (41.5%) and Bratislava (serogroup Australis) (33.8%). The authors suggested that the high seroprevalence and the detected serovars likely have a link between swine and human infection.

In the African continent, Potis et al. [45] collected blood samples from 5041 pigs aged approximately 16 to 24 weeks and reported an overall seropositivity of 22.18% (95% CI = 21.05–23.35%). The authors stated that the low seropositivity of serovar Pomona and the low titers of Icterohaemorrhagiae, Hardjo and Bratislava indicate that leptospirosis is not a major problem in swine in South Africa. It was possible to observe that there is a lack of information in the methodology, such as type of rearing, characteristics of the properties, climate of the region and time of year. The inclusion of these data could contribute to the understanding of the epidemiology of leptospirosis in the study region.

The Asian continent had the lowest combined seropositivity of 13.36% (95% CI = 8.32–20.75%). Boqvist et al. [30], in their serological research carried out in southern Vietnam, identified variations in the seropositivity of the serovars Bratislava and Icterohaemorrhagiae and this seropositivity was higher in a dry climate. The authors concluded that in regions where water was constantly abundant and pigs were bred extensive over the year, variations in the prevalence of leptospirosis were highly significant. Lee et al. [40] evaluated 1959 blood samples from fattening pigs in five provinces (Son La, Hanoi, Nghe An, Dak Lak and An Giang) and identified that the seropositivity of leptospirosis in sows (5.28%; 95% CI = 3.94–6.93%) was slightly higher than males (4.88%; 95% CI = 3.51–6.58%), but this difference was not statistically significant. Naito et al. [43] carried out a survey with 24 farms in Japan, and stated that antibiotics as food additives can interfere with the isolation of leptospires. Considering that some producers use antibiotics as a food additive and this use can prevent infection and decrease seropositivity values, studies that collect samples from pigs in the finishing or slaughter process may not identify the infectious agent due to recent exposure to antibiotics. In this sense, investigations of leptospirosis in swine should be strategically planned, comparing groups that received antibiotics with groups from the same region that did not, in order to elucidate whether antibiotics neutralize the pathogen and prevent leptospira infection.

Chadsuthi et al. [33] analyzed data on leptospirosis infection in humans and animal species (buffalo, cattle and pigs) during 2010 to 2015 in the region of Thailand. The seroprevalence was 23.7% in humans, 24.8% in buffaloes, 28.1% in cattle and 11.3% in pigs. The most prevalent serovars were Shermani, followed by Bratislava, Panamá and Sejroe in humans, Shermani, Ranarum and Tarassovi in buffaloes and Shermani and Ranarum in cattle and pigs. These findings reinforce the importance of carrying out surveys aimed at investigating all species susceptible to leptospiral infection, in order to favor the development of control and eradication strategies for the disease in livestock and in the environment.

In Oceania, all studies that were found in the search for this research were conducted in Australia. Chappel et al. [34] found serological evidence for the presence of *Leptospira interrogans* serovar Bratislava, and Chappel et al. [35] identified the seropositivity of *Leptospira interrogans* serovar Pomona, but the most recent survey identified on the continent was carried out by Ridoutt et al. [46] with wild pigs, with an increasing seropositivity of the serovar Pomona. These authors justified that the increase in the seropositivity of the serovar Pomona in the New South Wales region, in Australia, occurred in years preceded by floods and rodent pests, and that these risk factors were associated with the increased potential of zoonotic infection in the region.

In Europe, the combined seropositivity was 13.30% (95% CI = 6.40–25.62%). A total of 44,469 pigs were assessed and the main serogroups were Australis, Pomona, Icterohaemorrhagiae and Autumnalis. Bertoline et al. [29] carried out a serological survey with 1194 sera from 61 farms located in five different regions of northwest Italy, and samples were collected from healthy slaughtered pigs. The presence of antibodies against four serovars of *Leptospira* spp. was evaluated. Overall, 52.5% of the analyzed herds had at least one positive animal and 34.4% had at least one positive pig with a titer ≥1:400. A percentage of 16.6% of the sera were positive and 5.9% of the samples had a positive titer ≥1:400. Tuscany and Lombardy had the highest percentage of positive farms (64.3% and 54.6%, respectively), and sera (28.5% and 13.3%, respectively), probably due to environmental conditions and potential risk factors, which promote the maintenance and dissemination of leptospires in these areas. The main serogroups represented were Australis (21.3% positive farms, 8.2% positive sera) and Pomona (18.0% positive farms, 8.1% positive sera). In pigs, these serogroups are the most detected worldwide; however, these results seem to show the resurgence of the Pomona serogroup in pigs in the investigated areas. The authors suggest that porcine leptospirosis is probably underestimated in Italy and may pose a potential risk to human and animal health.

André-Fontaine [39], in the period of 1988 to 2007, analyzed serum samples from more than 40,000 cattle; 40,000 pigs; 20,000 horses; and 9500 dogs. Five serogroups of *Leptospira* spp. were highlighted, with specific variations within the four animal species: Icterohaemorrhagiae, Australis, Sejroe, Grippotyphosa and Autumnalis. The researcher reported that the seropositivity and incidence of each serogroup varied for each species over these almost 20 years, and that some serogroups appeared for a few years but then disappeared, and stated that these results served to reveal the complex epidemiological characteristics of leptospirosis in the continent of Europe.

Mousing et al. [42] evaluated a total of 796 sows and gilts from 30 Danish sow herds three times at 6-week intervals for serum antibodies to *Leptospira bratislava* by the microscopic agglutination technique (MAT) test. Of the 30 farms, 21 (70%) had consistently positive prevalences in sows and gilts of 4–13%. A high prevalence and cumulative low incidence of seroreactivity was demonstrated in first farrowing gilts, followed by a low prevalence and cumulative incidence from farrowing 2 to 3, and a high prevalence and cumulative incidence from farrowing 5.

The high heterogeneity observed in this review can be justified by the sampling methodology adopted by the researchers who did not perform the sample calculation based on probabilistic criteria, which can generate selection bias and, consequently, reflect on the prevalence. To reduce the risk of bias in observational studies, it is necessary to focus the efforts on standardizing methodologies based on probabilistic sampling, which can provide, through random selection, a small group of animals to represent the general population. This provides a greater reliability of the results and the generation of sufficient data to identify the epidemiological profile of the diseases that affect this population [50].

## 5. Conclusions

The results suggest that there is high seropositivity for leptospirosis in pigs worldwide. Information compiled from this research is relevant to understanding the spread of leptospirosis globally. It is expected that these indicators will contribute to a better understanding of the epidemiology of the disease with a focus on its control and, consequently, on the reduction of cases in the human and animal population.

## Figures and Tables

**Table 1:** Summary of the main characteristics of the 20 studies included in the meta-analysis of seropositivity of swine leptospirosis.

References	Country	Continent	Sample Size	No. of Positive Animals	MAT Cut-Off	Characteristics of the Study Population
Azevedo, S.S. et al. [28]	Brazil	South America	131	44	1:100	Pigs from small rural properties; blood samples collected at slaughter in the public slaughterhouse in the municipality of Patos, northeast region of Brazil.
Bertelloni, F. et al. [29]	Italy	Europe	1194	198	1:100	Healthy pigs from 61 farms located in five different regions of northwest Italy; samples collected at slaughter.
Boqvist, S. et al. [30]	Vietnam	Asia	429	121	1:100	Sows from five smallholder state farms in the Mekong delta, southern Vietnam; blood samples collected during farm visits.
Buchholz, A.E. et al. [31]	USA	North America	804	277	1:100	Feral pigs on the islands of Oahu, Hawaii, Kauai and Maui; blood samples collected opportunistically by hunters and wildlife biologists.
Calderón, A. et al. [32]	Colombia	South America	383	214	1:100	Pigs from 18 farms in the middle region of the Sinú River in the department of Córdoba; blood samples collected during farm visits.
Chadsuthi, S. et al. [33]	Thailand	Asia	3138	356	1:100	Serum samples from pigs, mostly from rural areas, Thailand.
Chappel, R.J. et al. [34]	Australia	Oceania	86	21	1:512	Growing pigs, originating from 49 farms; blood samples collected at slaughter.
Chappel, R.J. et al. [35]	Australia	Oceania	368	42	1:1024	Serum samples obtained from pigs from 42 farms and slaughtered in three slaughterhouses.
Chappel, R.J. et al. [36]	Australia	Oceania	10,440	383	1:512	Blood samples collected from pigs at slaughter.
Chatfield, J. et al. [37]	USA	North America	324	107	1:100	Blood samples collected from feral hogs killed at managed hunts and by permitted trappers throughout Florida.
Figueiredo, Í.L. et al. [38]	Brazil	South America	126	18	1:100	Pigs from small rural properties; blood samples collected at slaughter in the public slaughterhouse in the municipality of Patos, northeast region of Brazil.
André-Fontaine, G. [39]	France	Europe	42,479	11,265	1:100	Porcine sera tested from 1988 to 2007 from the Leptospira Medical and Molecular Bacteriology Laboratory of the Nantes National College of Veterinary Medicine, Food Science and Engineering.
Lee, H.S. et al. [40]	Vietnam	Asia	1949	160	1:100	Blood samples from fattening pigs were randomly collected slaughterhouses in five provinces (Son La, Hanoi, Nghe An, Dak Lak and An Giang). The selected provinces represented the different ecological and climatic conditions zones in Vietnam.
Leite, A.I. et al. [41]	Brazil	South America	412	324	1:100	Pigs originating from 20 properties in the county of Mossoró, state of Rio Grande do Norte, Brazil.
Mousing, J. et al. [42]	Denmark	Europe	796	36	1:100	Sows and gilts from 30 Danish sow farms.
Naito, M. et al. [43]	Japan	Asia	938	106	1:100	Fattening piglets from 24 farms in Hokkaido, Kagoshima and Okinawa prefectures in Japan in 2001–2005.
Petrakovsky, M.J. et al. [44]	Argentina	South America	3631	1102	1:100	Domestic pigs. Assignment of properties and samples was carried out in proportion to those registered in each province across the country.
Potis, A.D. et al. [45]	South Africa	Africa	5041	1118	1:50	Blood samples from slaughtered pigs from 341 facilities were randomly collected from slaughterhouses. Between ten and twenty samples were taken from each of the facilities.
Ridoutt, C. et al. [46]	Australia	Oceania	239	133	1:100	During 2012 and 2013, serum samples were collected from feral pigs in New South Wales.
Valença, R.M.B. et al. [47]	Brazil	South America	342	55	1:100	Pigs from farms located in the state of Alagoas, Brazil.

**Table 2:** Summary of the meta-analysis of the seropositivity of swine leptospirosis according to continent and sample size.

Subgroup	Number of Studies	Sample Size	No. of Positive	Combined Prevalence (95% CI)	Heterogeneity	
					*p*	I^2^
**Combined overall prevalence**	**20**	**73,250**	**16,080**	**21.95% (16.65–28.37%)**	**<0.01**	**99.45%**
**Continent**						
Europe	3	44,469	11,499	13.30% (6.40–25.62%)	<0.01	98.99%
South America	6	5025	1757	36.40% (20.36–56.16%)	<0.01	98.84%
Asia	4	6454	743	13.36% (8.32–20.75%)	<0.01	97.60%
Africa	1	5041	1118	22.18% (21.05–23.35%)	Não aplicável	
North America	2	1128	384	34.05% (31.34–36.86%)	0.65	0%
Oceania	4	11,133	579	17.40% (3.15–57.70%)	<0.01	99.56%

## Data Availability

The datasets generated during and/or analyzed during the current study are available from the corresponding author upon request.
